# Mesenchymal stem cell-derived microparticles ameliorate peritubular capillary rarefaction via inhibition of endothelial-mesenchymal transition and decrease tubulointerstitial fibrosis in unilateral ureteral obstruction

**DOI:** 10.1186/s13287-015-0012-6

**Published:** 2015-03-11

**Authors:** Hoon Young Choi, Hyun Gyu Lee, Beom Seok Kim, Sun Hee Ahn, Ara Jung, Mirae Lee, Jung Eun Lee, Hyung Jong Kim, Sung Kyu Ha, Hyeong Cheon Park

**Affiliations:** Department of Internal Medicine, Gangnam Severance Hospital, Yonsei University College of Medicine, Seoul, Korea; Severance Institute for Vascular and Metabolic Research, Yonsei University College of Medicine, Seoul, Korea; Department of Microbiology and Immunology, Yonsei University College of Medicine, Seoul, Korea; Department of Internal Medicine, Severance Hospital, Yonsei University College of Medicine, Seoul, Korea; Department of Internal Medicine, Yong-In Severance Hospital, Gyeongi-do, Korea; Department of Internal Medicine, CHA Bundang Medical Center, CHA University, Gyeongi-do, Korea

## Abstract

**Introduction:**

Microparticles (MPs) derived from kidney-derived mesenchymal stem cells (KMSCs) have recently been reported to ameliorate rarefaction of peritubular capillaries (PTC) in ischemic kidneys via delivery of proangiogenic effectors. This study aimed to investigate whether KMSC-derived MPs show anti-fibrotic effects by ameliorating endothelial-to-mesenchymal transition (EndoMT) in human umbilical vein endothelial cells (HUVEC) *in vitro* and by preserving PTC in kidneys with unilateral ureteral obstruction (UUO) *in vivo*.

**Methods:**

MPs isolated from the supernatants of KMSC were co-cultured with HUVEC to assess their *in vitro* biologic effects on endothelial cells. Mice were treated with MPs via the tail vein after UUO injury to assess their anti-fibrotic and PTC sparing effects. Renal tubulointerstitial damage and inflammatory cell infiltration were examined with Masson’s trichrome, F4/80 and α-smooth muscle actin (α-SMA) staining and PTC rarefaction index was determined by CD31 staining.

**Results:**

KMSC-derived MPs significantly ameliorated EndoMT and improved *in vitro* proliferation of TGF-β1 treated HUVEC. *In vivo* administration of KMSC-derived MPs significantly inhibited EndoMT of PTC endothelial cells and improved PTC rarefaction in UUO kidneys. Furthermore, administration of KMSC-derived MPs inhibited inflammatory cell infiltration as well as tubulointerstitial fibrosis in UUO mice as demonstrated by decreased F4/80 and α-SMA-positive cells and Masson’s trichrome staining, respectively.

**Conclusions:**

Our results suggest that KMSC-derived MPs ameliorate PTC rarefaction via inhibition of EndoMT and protect against progression of renal damage by inhibiting tubulointerstitial fibrosis.

## Introduction

Unilateral ureteral obstruction (UUO) is a well-established *in vivo* model of tubulointerstitial scarring. It involves virtually all renal intrinsic and infiltrating cells and is characterized by alterations in their phenotype and accumulation of excessive extracellular matrix proteins [1-4]. Another histologic alteration frequently noted in UUO is rarefaction of peritubular capillaries (PTC) that are essential for providing nutrients and oxygen to the surrounding tubules and interstitial cells [5,6]. Renal microvasculature injury leading to PTC rarefaction and resulting in chronic tissue hypoxia is a major contributor to renal disease progression [7]. Recently, myofibroblasts have been shown to rise from endothelial cells via endothelial-to-mesenchymal transition (EndoMT) induced by the transforming growth factor-β (TGF-β) family of regulatory polypeptides in experimentally induced fibrotic diseases. Taken together, PTC rarefaction derived via EndoMT may play an important role in the process of kidney fibrosis in UUO [8].

We previously demonstrated that kidney-derived mesenchymal stem cells (KMSCs) are capable of homing to injured renal tubulointerstitium after acute ischemic-reperfusion injury and inducing tissue repair via secretion of proangiogenic factors, such as vascular endothelial growth factor (VEGF)-A. Administration of MSCs prevented the loss of PTC possibly due to local production of growth factors, rather than by differentiation into renal cells, and the maintenance of interstitial vasculature was associated with less interstitial fibrosis [9]. The paracrine actions of MSC administration were recently demonstrated to involve the release of microparticles (MPs) by MSCs. These MSC-derived MPs play important roles in cell-to-cell communication via transportation of various mRNA or proteins and interact via specific receptor ligands to exert their protective effects [10-12]. In a previous study, KMSC-derived MPs delivered proangiogenic signals and contributed to recovery of renal function in acute ischemia-reperfusion injury [13]. MSC-derived MPs afforded renoprotective effects in various models of acute kidney injury by ameliorating apoptosis of tubular epithelial cell and stimulating tubular epithelial cell proliferation [10,14]. However, studies have yet to demonstrate the efficacy of KMSC-derived MPs in preventing renal fibrosis and PTC rarefaction in an *in vivo* model of tubulointerstitial scarring.

In this study, we assessed the effect of KMSC-derived MPs on the development of renal fibrosis in a murine model of UUO. Moreover, we investigated the mechanism by which KMSC-derived MPs exert their PTC protective effects, focusing on EndoMT.

## Methods

### Culture of mouse kidney mesenchymal stem cells and isolation of microparticles

We previously isolated and cloned a fibroblast-like cell line from the kidneys of adult FVB/N mice [15]. These KMSCs were cultured on gelatin-coated dishes in minimum essential medium (MEM) with 10% horse serum (Gem Biotech, Woodland, CA, USA) as previously described [15]. For generation of MPs, culture medium was replaced with serum free alpha MEM, and KMSCs were then placed in a hypoxic chamber (<1% O2) for 24 hours. Cell debris was removed by centrifugation at 1,000 g for 10 minutes at room temperature. The cell-free supernatants were centrifuged at 50,000 g (Beckman Coulter Optima L-90 K ultracentrifuge) for two hours at 4°C and washed in phosphate-buffered saline (Sigma, St Louis, MO, USA) with a second centrifugation under the same conditions. The supernatants collected from the second ultracentrifugation washing (‘Vehicle control’) were used for *in vitro* experiments, such as EndoMT and proliferation assay of TGF-β1-treated human umbilical vein endothelial cells (HUVECs).

Thereafter, MPs from KMSCs were labeled with PKH26 dye (Sigma) or cell-tracker (Invitrogen, Carlsbad, CA, USA) for tracing *in vivo* and *in vitro* experiments. MPs were identified by fluorescence microscopy (Carl Zeiss, Göttingen, Germany) or fluorescence-activated cell sorting (FACS) analysis (BD Canto II, Franklin Lakes, NJ, USA) and electron microscopy (JEM-1011, Tokyo, Japan) as previously described [13].

To abolish mRNA-dependent effects, MPs were preincubated with 1 U/ml RNase (Ambion Inc., Austin, TX, USA) for one hour at 37°C; the reaction was stopped by addition of 10 U/ml RNase inhibitor (Ambion Inc.).

The protein content of MPs was quantified by the Bradford method (BioRad, Hercules, CA, USA).

### *In vitro* cell proliferation assay and TGF-β-induced endothelial-to-mesenchymal transition in cultured human umbilical vein endothelial cells

HUVECs were purchased from Lonza (Walkersville, MD, USA) and cultured on endothelial growth medium (EGM) (Lonza) with 20% fetal bovine serum (FBS; Lonza) and 1% penicillin/streptomycin (Gibco, Carlsbad, CA, USA). Cells between passages two and six were used. To test cell viability, we used an EZ-Cytox Cell Viability Assay Kit (Daeil Lab Service Co, Seoul, Korea). The assay is based on the cleavage of tetrazolium salt to water-soluble formazan by the succinate-tetrazolium reductase system which belongs to the respiratory chain of the mitochondria and is active only in viable cells. Therefore, the amount of the formazan dye is directly proportional to the number of living cells. HUVEC were seeded at 5,000 cells/well into 96-well plates in EBM-2 media (Lonza) deprived of FBS. After 36 hours, we added 20 μl of EZ-Cytox kit reagent into each cell cultured well of a 96-well microplate and incubated at 37°C, in 5% CO2 incubator for 90 minutes. Cell viability at baseline was measured using a microplate reader at 450 nm.

Recombinant TGF-β1 was used at a concentration of 5 ng/ml as previously reported [16] for *in vitro* cell proliferation assay and EndoMT experiments in cultured HUVEC. KMSC-derived MPs (20 ug for i*n vitro* cell proliferation assay, 1 to approximately 2 × 10^7^/well for EndoMT) were added to each well.

### Western blot analysis

For *in vitro* cellular experiments, cells were lysed with radioimmunoprecipitation assay (RIPA) buffer (20 mM Tris, pH 7.8, 140 mM NaCl, 1 mM ethylenediaminetetraacetic acid (EDTA), 1% Triton X-100, 0.1% SDS, 1% sodium deoxycholate, 1 mM NaF, and 1 mM orthovanadate) with Complete Mini protease inhibitors (Roche, Indianapolis, IN, USA). Protein concentration was determined using a Bradford assay (Bio-Rad). Equal amounts of protein were separated in 8% to 12% SDS-PAGE gels and transferred to Immobilon-P membranes (Millipore, Bedford, MA, USA). Following blocking with TBS/5% nonfat dry milk, membranes were incubated with the following primary antibodies: anti-α-SMA (1:1,000; Sigma), β-actin (1:1,000; Cell Signaling, Danvers, MA, USA), and anti-CD31 (1:2,000; Abcam, Cambridge, UK). Following washes with TBS/0.1% Tween 20, membranes were incubated with horseradish peroxidase-conjugated secondary antibodies (Santa Cruz, Dallas, TX, USA) for 60 minutes at room temperature. The analysis was repeated in triplicate to ensure the reproducibility of results. Membranes were washed and protein was detected by chemiluminescence.

The intensity of the band of CD31 and α-smooth muscle actin (α-SMA) proteins from western blots was quantified by using NIH Image J 1.34 s software and normalized to β-actin.

### Mouse model of unilateral ureteral obstruction

The animal study protocol was designed in accordance with the guidelines for use of laboratory animals and approved by the Institutional Animal Care and Use Committee of Yonsei University Healthcare System (IACUC approval number: 2010-0302-1). Adult (eight to ten weeks old) FVB/N mice were purchased from The Koatech (Gyeonggi-Do, Korea). Animals were kept under temperature-controlled conditions with a 12-hour light/dark cycle, with water and food *ad libitum*. The experiments were designed to test the hypothesis that exogenous administration of MSC-derived MPs would ameliorate progressive tubulointerstitial scarring and PTC loss, characteristic of UUO models. UUO was performed using an established protocol [17]. Briefly, mice were anesthetized with intraperitoneal injection of a combination of Zoletil (20 mg/kg) and xylazine (10 mg/kg) and placed on a heated surgical thermo plate (Jeung Do Bio & Plant Co, Seoul, Korea). The left ureter was visualized via a flank incision and ligated with 3-0 silk at two points just below the lower pole of the left kidney; the wound was closed in layers. Following UUO, mice were randomly divided into four experimental groups (n = 5) and administered the following agents via the tail vein: vehicle (saline) only, KMSCs (1 × 10^6^ per mouse), KMSC-derived MPs (2 × 10^7^ per mouse), or RNase treated MPs. In each group, mice were sacrificed at day 7 after UUO (n = 5 per group). The kidneys were subsequently removed and renal tissues were analyzed for localization of MPs and markers of renal fibrosis or PTC rarefaction.

### Immunohistochemical and immunofluorescent analysis

For analysis of kidney fibrosis, kidney sections were stained with Masson’s trichrome and anti-α-SMA (Sigma). Positive areas of Masson’s trichrome and α-SMA staining were evaluated in relation to the unit area and expressed as a percentage per unit area using MetaMorph microscopy image analysis software (Molecular Devices, Sunnywale, CA, USA). Microscopic assessment was carried out in a blinded manner and 20 randomly selected fields from each slide section were examined at ×400 magnification.

Immunohistochemistry for detection of proliferation and rarefaction of PTC was performed as described previously [18]. Kidney sections were labeled with anti-CD31 (Santa Cruz) and anti-PCNA (proliferating cell nuclear antigen, Millipore). Immunoperoxidase staining was performed using a 1:100 dilution of anti-rabbit horseradish peroxidase (HRP, Abcam) or anti-mouse IgM (Invitrogen). Scoring for CD31 and PCNA-positive cells was determined by counting the number of positive nuclei per high-power field (HPF) in 10 randomly chosen fields through the kidney specimens. Quantification of PTC loss was performed by calculating the rarefaction index as previously described [18]. Briefly, the CD31-immunostained sections were examined across a 10 × 10 grid under a × 40 objective. Each square within the grid that did not contain CD31-positive capillaries was counted. At least 20 fields in the cortex and outer medulla were examined on the cross-section of each kidney, and a mean score per section was calculated. This scoring system, thus, inversely reflects PTC rarefaction, whereby low values represent intact capillaries and higher values indicate loss of capillaries (the minimum possible capillary score is 0, and the maximum score is 100).

Immunohistochemistry for macrophages was confirmed using anti-F4/80 (Abcam). The quantification of F4/80-positive cells was expressed as number of cells per HPF [19].

Colocalization of CellTracker™-labeled MPs within the peritubular interstitium was demonstrated by detecting green fluorescent CellTracker™ and the presence of red fluorescent CD31 (Santa Cruz) in kidney sections by confocal microscopy (LSM 780, Carl Zeiss, Jena, Germany) using × 40 fluorescence objective lens and analyzed with LSM5 software.

The detection of EndoMT was performed as previously described [20]. Kidney sections were labeled with anti-CD31 (Santa Cruz) and anti-α-SMA (Sigma). The secondary antibodies included Alexa Fluor 488- (Jackson, West Grove, PA, USA) and Cy3–(Jackson) conjugated secondary antibodies. At least 15 randomly selected fields were analyzed for co-localization of endothelial and fibroblast markers by confocal microscopy (LSM 780, Carl Zeiss, Jena, Germany).

To detect cell apoptosis in the UUO kidney, terminal deoxynucleotidyl transferase-mediated dUTP nick end-labeling (TUNEL) assays were performed using an *In Situ* Cell Death Detection Kit (Roche, Indianapolis, IN, USA). Positive nuclei in the field were examined under a confocal microscope (LSM 780, Carl Zeiss, Jena, Germany) using a × 40 fluorescence objective lens and analyzed with LSM5 software. Twenty randomly selected fields from the cortex and corticomedullary junction on each slide section were examined as previously reported [13].

### Statistical analysis

Data are expressed as mean ± s.e.m. Differences between the groups were analyzed by analysis of variance or the Kruskal–Wallis test using SPSS software version 20.0 (SPSS, Chicago, IL, USA). All *P*-values less than 0.05 were considered statistically significant.

## Results

### KMSC-derived MPs attenuate TGF-β1-induced EndoMT of HUVEC *in vitro*

**Figure 1:**
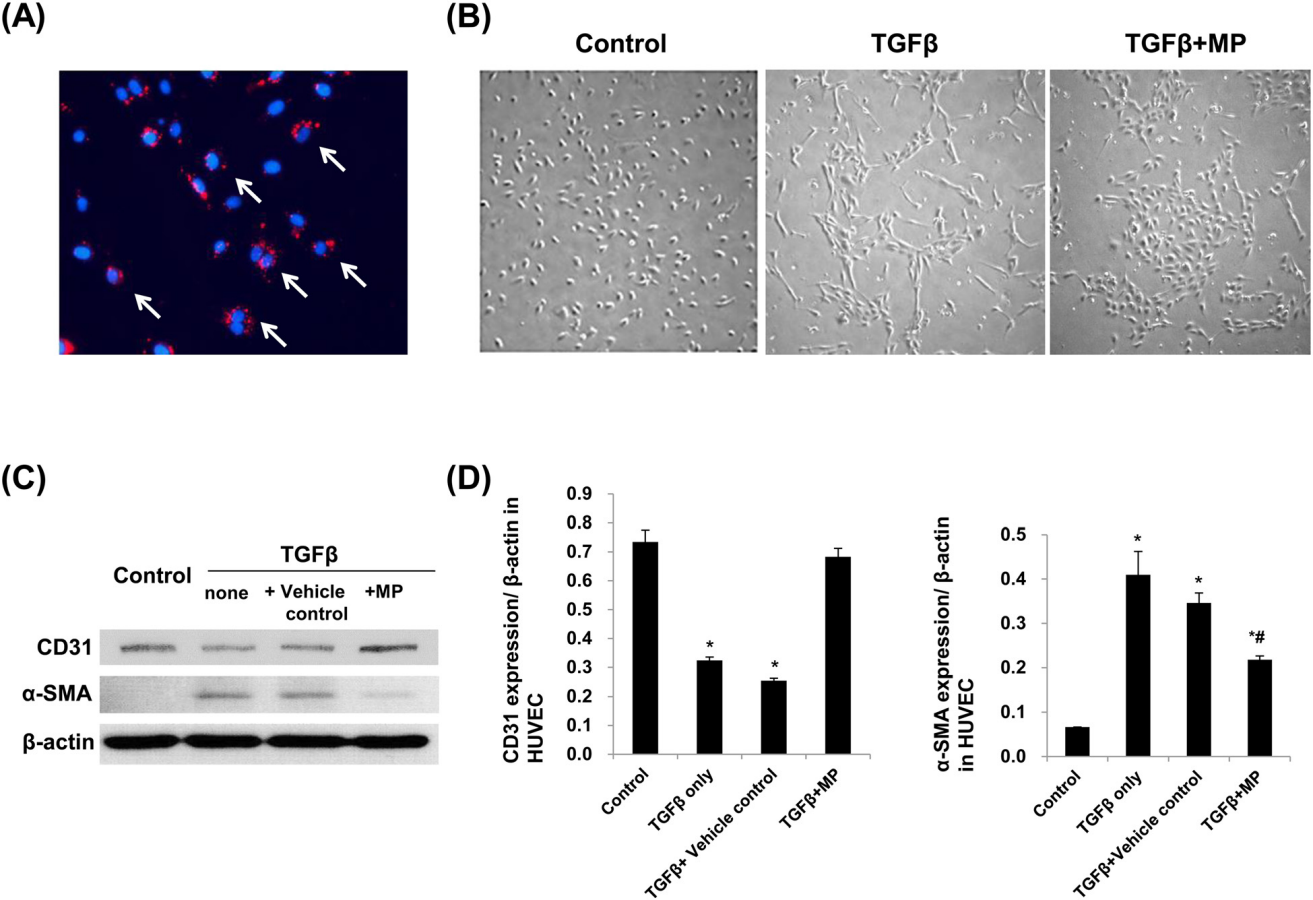
**Incorporation of PKH26-labeled MPs into cultured HUVEC and the** ***in vitro*** **effect of MPs on endothelial-to-mesenchymal transition.** Immunofluorescence microscopy revealed that red PKH26-labeled MPs were readily incorporated into cultured HUVEC, as shown by DAPI staining **(A)**, white arrows). Light microscopy analysis shows the morphologic changes of HUVEC treated with TGF-β1 alone or with TGF-β1 and KMSC-derived MPs simultaneously, compared to non-treated control cells (‘Control’) **(B)**. Western blot analysis shows CD31 and α-SMA protein expression levels in HUVEC treated with vehicle control or KMSC-derived MPs. **(C)**. A bar graph shows the quantification of CD31 and α-SMA protein expression levels normalized by β-actin protein expression measured using the Image J program **(D)**. Non-treated control was expressed as ‘Control’. Vehicle control of MPs comprised MP-free supernatants after centrifugation was expressed as ‘Vehicle control.’ Results are expressed as the mean ± SE of three different experiments. The Kruskal-Wallis test was performed; * *P* <0.05 versus non-treated control. # *P* <0.05 versus TGF-β1 alone. DAPI, 4',6-diamidino-2-phenylindole; HUVEC, human umbilical vein endothelial cells; KMSC, kidney-derived mesenchymal stem cells; MPs, microparticles; SE, standard error; TGF-β1, transforming growth factor- β1; α-SMA, α-smooth muscle actin.

First, we confirmed the incorporation of MPs which were pre-incubated with PKH26 dye for 30 minutes at 37°C into cultured HUVEC by immunofluorescence microscopy analysis (Figure 1A). To investigate the effects of KMSC-derived MPs on EndoMT, cultured HUVEC were treated with a predetermined concentration of TGF-β1. We found that TGF-β1 treatment in cultured HUVEC led to morphological changes into a fibroblast-like spindle-shaped form compared to the cobble stone appearance of non-treated controls, and these changes were inhibited by KMSC-derived MPs (Figure 1B). Next, we checked CD31 and α-SMA protein expression levels in HUVEC treated without or with TGF-β1. Western blot analysis revealed significant decreases in CD31 protein expression in HUVEC treated with TGF-β1, compared to non-treated control cells (CD31/β-actin: 0.32 ± 0.01 *versus* 0.73 ± 0.04, *P* <0.05), while incubation with KMSC-derived MPs inhibited this effect (CD31/β-actin: 0.68 ± 0.03, *P* <0.05 *versus* TGF-β1 treatment). Additionally, TGF-β1 treatment significantly increased α-SMA protein expression in HUVEC compared to non-treated control cells (α-SMA/β-actin: 0.41 ± 0.05 *versus* 0.07 ± 0.00, *P* <0.05). This effect was significantly attenuated by treatment with KMSC-derived MPs (α-SMA/β-actin: 0.22 ± 0.00 *versus* TGF-β1 treatment). Vehicle control which was MP-free supernatants had no effect on the EndoMT induced by TGF-β1 treatment (Figure 1C, D).

### *In vitro* proliferative effects of KMSC-derived MP on HUVEC

**Figure 2:**
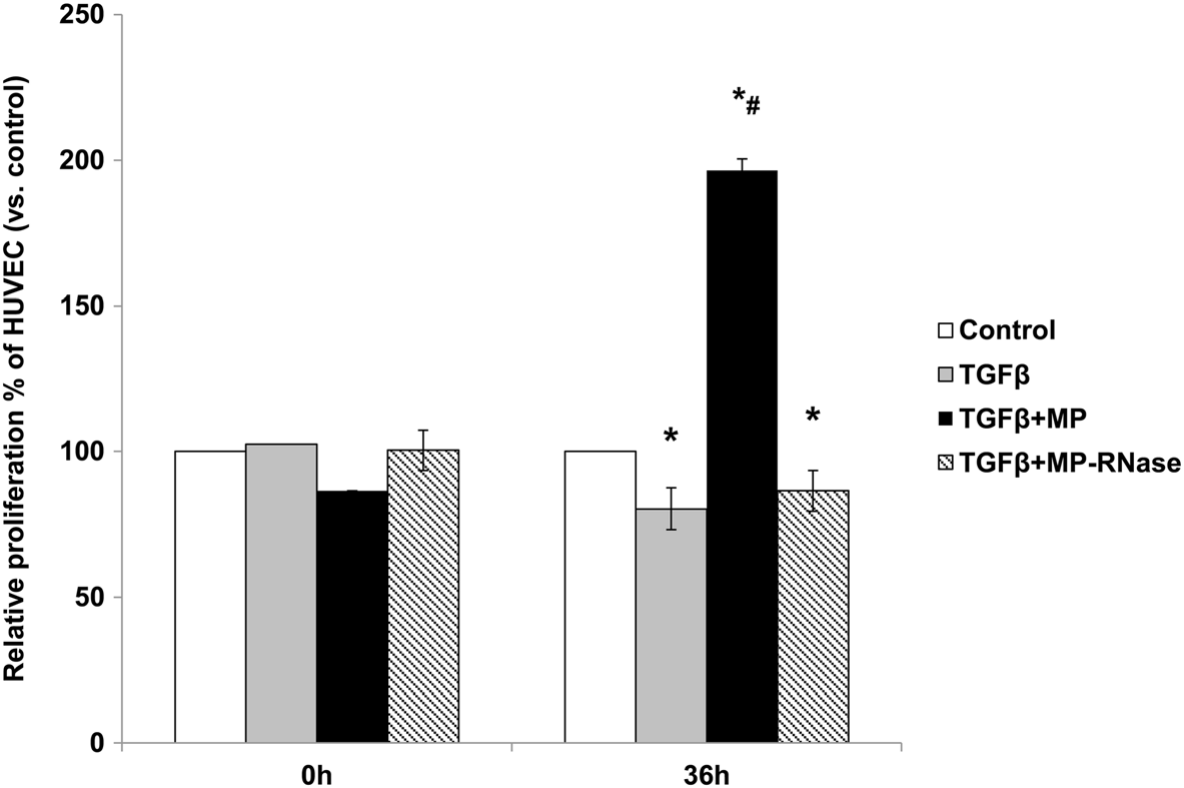
***In vitro*** **cell proliferation effects of MPs.** Cell proliferation of HUVEC non-pretreated control (‘Control’), pretreated with TGF-β1 alone, with TGF-β1 treatment simultaneously with KMSC-derived MP, and MPs preincubated with RNase. Non-treated control was expressed as ‘Control.’ Results are expressed as the mean ± SE of three different experiments. The Kruskal-Wallis test was performed; * *P* <0.05 versus non-treated control. # *P* <0.05 versus TGF-β1 alone. HUVEC, human umbilical vein endothelial cells; KMSC, kidney-derived mesenchymal stem cells; MPs, microparticles; SE, standard error; TGF-β1, transforming growth factor- β1.

We further evaluated the *in vitro* proliferative effects of KMSC-derived MPs on HUVEC. Incubation of HUVEC with TGF-β1 significantly reduced the proliferation of HUVEC compared to non-treated control cells. KMSC-derived MPs significantly improved the proliferation of HUVEC in serum deprived culture conditions, compared to non-treated control cells, or TGF-β1 treated cells (relative degree of proliferation compared to non-treated control cells; KMSC-MPs 196.5 ± 4.7 *versus* TGF-β1 80.3 ± 4.0%, *P* <0.05). KMSC-derived MPs that were preincubated with RNase (MP-RNase) failed to demonstrate this beneficial effect on cell proliferation in cultured HUVEC (Figure 2).

### *In vivo* engraftment of MPs into PTC in UUO kidneys

**Figure 3:**
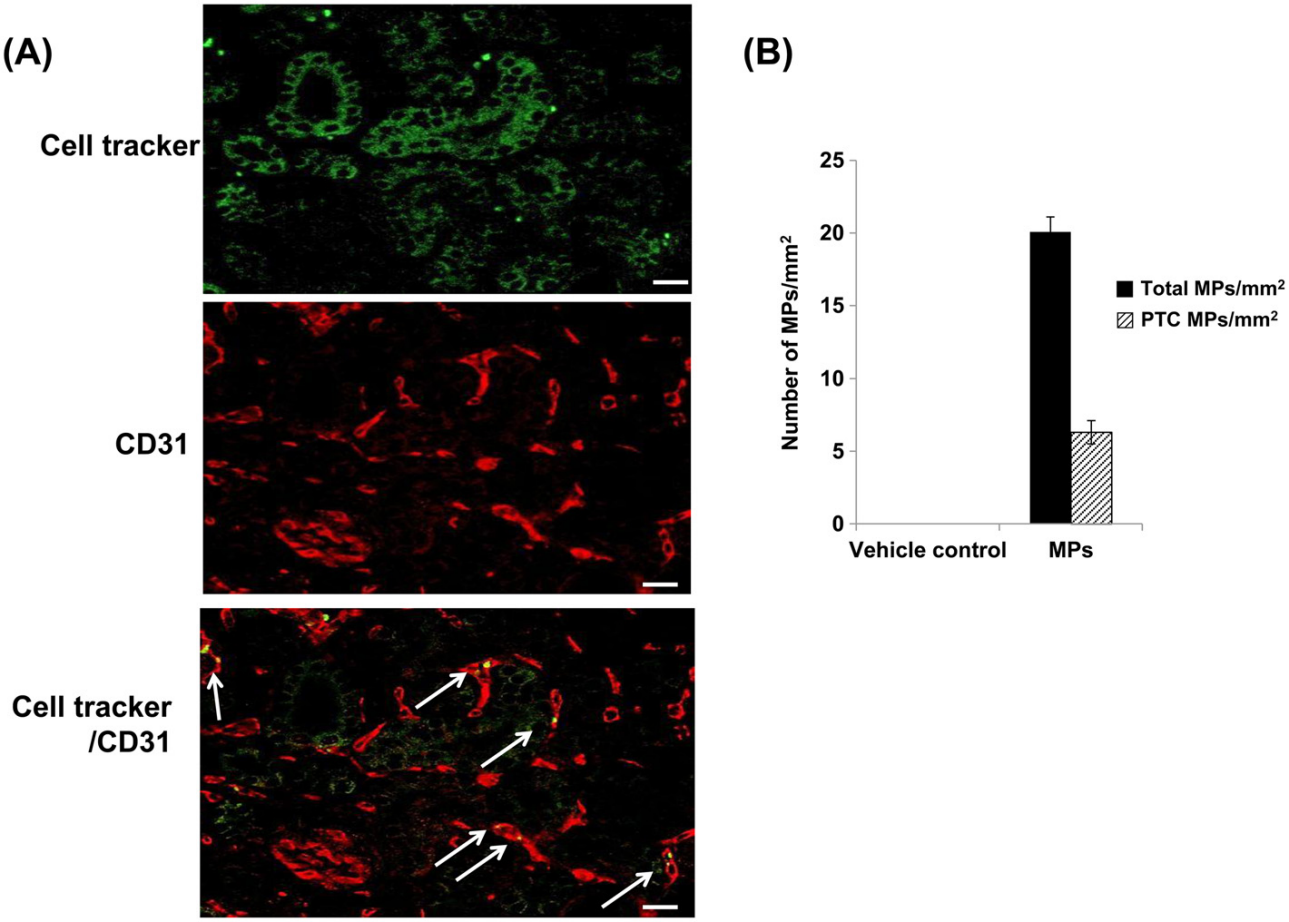
***In vivo*** **engraftment of MPs in UUO kidneys.** Confocal laser microscopy confirms peritubular capillaries stained with anti-CD31 and the engraftment of green CellTracker™ labeled MPs in peritubular capillaries. White bar represents 20 μm. White arrows indicate the engraftment of green CellTracker™ labeled MPs into peritubular capillaries **(A)**. Semi-quantitative analysis shows the number of engrafted MPs in UUO kidneys (‘Total MPs’) or within the peritubular capillaries (‘PTC MPs’) **(B)**. MPs, microparticles; UUO, unilateral uretal obstruction.

Next, we investigated the engraftment of KMSC-MPs in UUO kidneys. The green CellTracker™ labeled MPs were injected into FVB/N mice via the tail vein immediately after UUO and mice were sacrificed at day 7 after KMSC-MP injection. The green CellTracker™ labeled MPs were visualized in the UUO kidneys. These labeled MPs were also observed within the PTC expressing CD31 in confocal microscopy analysis (Figure 3A). Semi-quantitative analysis revealed that the average number of engrafted MPs in the UUO kidney (‘Total MPs’) or within the PTC (‘PTC MPs’) was 20.1 ± 1.0 MPs per mm^2^ and 6.3 ± 0.8 MPs per mm^2^, respectively (Figure 3B). No labeled MPs were detected in contralateral kidneys (data not shown).

### KMSC-derived MPs ameliorate tubulointerstitial fibrosis in UUO kidney

**Figure 4:**
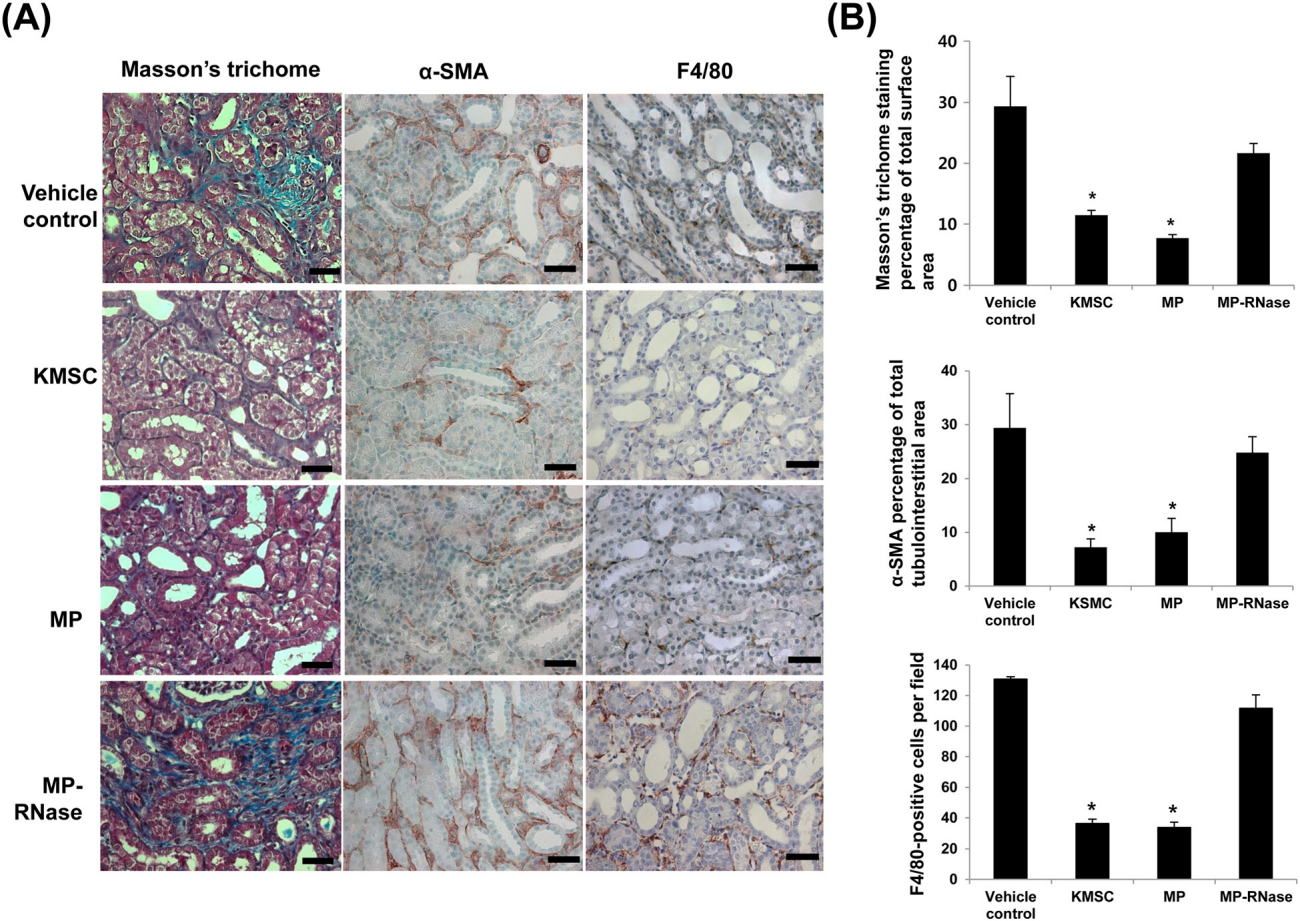
**Anti-fibrotic effects of KMSC or KMSC-derived MPs in UUO kidneys.** Light-field microscopic analysis of Masson’s trichrome–stained sections of UUO kidneys treated with vehicle control, KMSC, KMSC-derived MPs, or MP-RNase **(A)**. Quantitative analysis of tubulointerstitial fibrosis in Masson’s trichrome–stained sections using the Image J program (**B**, upper panel). Immunohistochemical study for α-SMA expression in UUO kidneys treated with vehicle control, KMSC, KMSC-derived MPs, or MP-RNase **(A)**. Quantitative analysis of tubulointerstitial α-SMA–positive area sections using the Image J program (**B**, middle panel). Immunohistochemical study for F4/80 positive cells in UUO kidneys treated with vehicle control, KMSC, KMSC-derived MPs, or MP-RNase **(A)**. Quantification analysis of F4/80 positive cells per field (**B**, lower panel). Ten randomly selected high-power fields were quantified and averaged to obtain the value for each mouse (*n =* 6 for each experimental group). Results are expressed as the mean ± SE of three different experiments. The Kruskal-Wallis test was performed; * *P* <0.05 versus vehicle control. Black bar represents 50 μm. KMSC, kidney-derived mesenchymal stem cells; MPs, microparticles; SE, standard error; UUO, unilateral uretal obstruction; α-SMA, α-smooth muscle actin.

To explore the anti-fibrotic effect of KMSC-derived MPs, we examined kidney sections stained with Masson’s trichrome and α-SMA in UUO kidneys treated with KMSC, KMSC-derived MPs, vehicle and MP-RNase. At day 7 after UUO injury, Masson’s trichrome staining showed more fibrotic lesions in the UUO kidneys injected with vehicle control. In contrast, KMSC or KMSC-derived MP injected mice kidneys showed less fibrosis in tubulointerstitial areas compared to kidneys injected with vehicle control or RNase-treated MPs (11.5 ± 0.8, 7.7 ± 0.6 *versus* 29.4 ± 4.8, 21.6 ± 1.6% of total tubulointerstitial area, respectively, *P <0.05) (Figure 4A, B). The kidney sections stained with α-SMA demonstrated similar findings as those for Masson’s trichrome staining. The UUO mice injected with vehicle control or MP-RNase showed increased α-SMA staining compared to that for UUO mice treated with KMSC or KMSC-derived MPs (29.4 ± 14.3, 24.8 ± 6.7 *versus* 7.2 ± 3.5, 10.0 ± 5.8% of total tubulointerstitial area, respectively, *P <0.05) (Figure 4A, B).

Next, we investigated the anti-inflammatory effects of KMSC or KMSC-derived MPs on UUO kidney. Immunohistochemistry for macrophages using F4/80 antibody confirmed increased macrophage infiltration in the tubulointerstitial area in UUO kidney. The number of F4/80-positive cells per HPF was counted in 10 randomly chosen sections. At seven days after UUO injury, the F4/80-positive cells were increased in mice kidneys treated with vehicle control. Administration of KMSC or KMSC-derived MPs reduced F4/80-positive cells compared to administration of vehicle control or RNase-treated MPs (36.3 ± 2.9, 33.8 ± 3.4 *versus* 130.8 ± 1.5, 111.7 ± 15.2 per field, respectively, *P <0.05) (Figure 4A, B).

**Figure 5:**
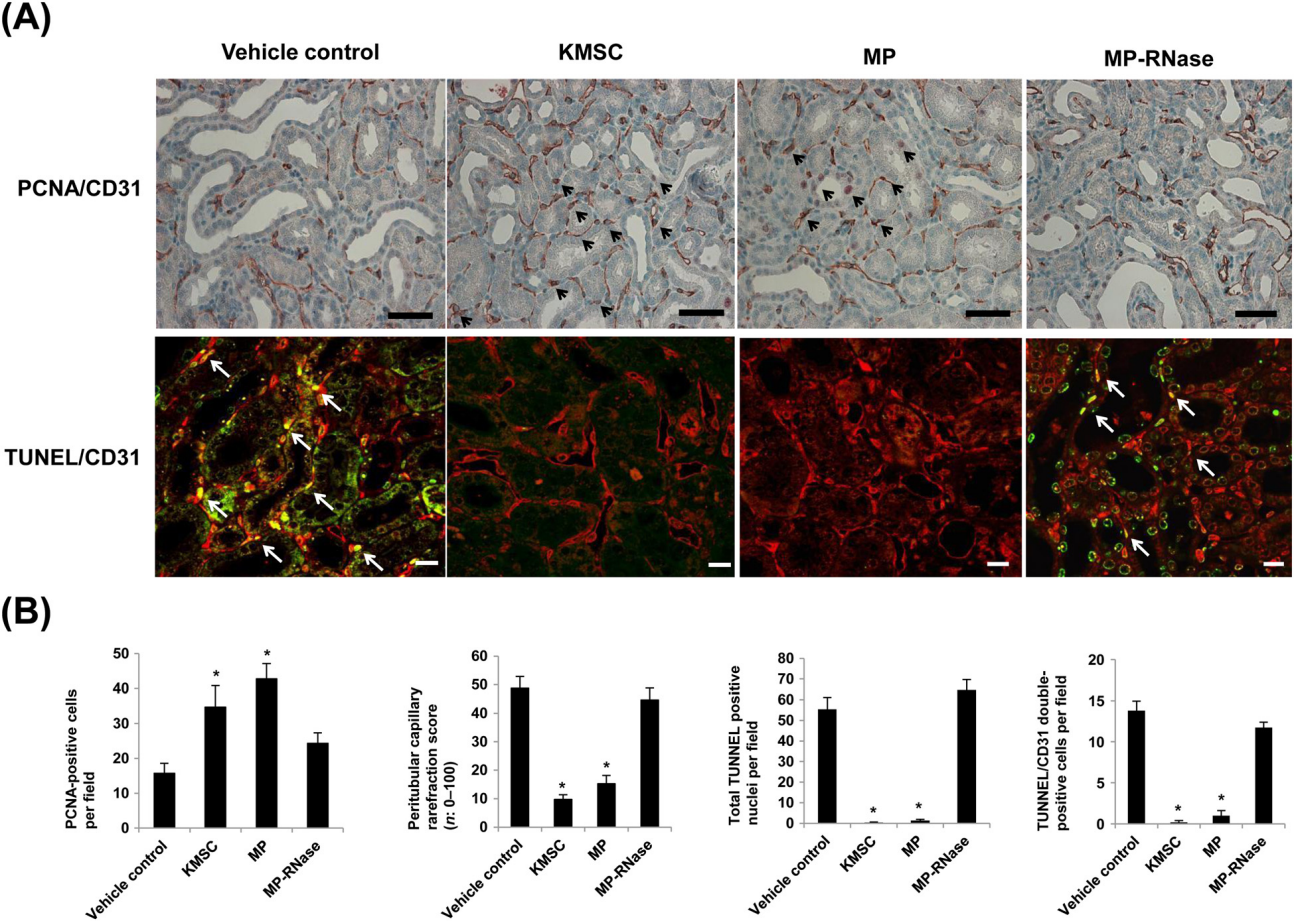
**Proliferation, microvascular rarefaction, and apoptosis in UUO kidneys.** Immunohistochemistry analysis showed representative images of CD31 and PCNA staining in UUO kidneys treated with vehicle control, KMSC, KMSC-derived MP, and MP-RNase (**A**, upper panel, CD31; brown color, PCNA; red color). Quantitative analysis of PCNA-positive cells (black arrows) per field in UUO kidneys. The PCNA-positive counting was used to assess the proliferation of tubular epithelial cells and peritubular capillaries **(B)**. Quantitative analysis of PTC rarefaction index was determined by CD31 staining **(B)**. Representative images of TUNEL and CD31 staining in UUO kidneys by confocal laser microscope analysis (**A**, lower panel, CD31; red color, TUNEL; green color). Quantitative analysis demonstrates the total number of TUNEL-positive nuclei and both TUNEL and CD31-positive PTC nuclei (white arrows) in UUO kidneys injected with KMSCs and KMSC-derived MPs, compared to those injected with vehicle control or MP-RNase. Results are expressed as the mean ± SE of six different experiments. The Kruskal-Wallis test was performed; * *P* <0.05 versus vehicle control **(B)**. Black bar represents 50 μm. White bar represents 20 μm. KMSC, kidney-derived mesenchymal stem cells; MPs, microparticles; PCNA, proliferating cell nuclear antigen; PTC, peritubular capillaries; SE, standard error; TUNEL, terminal deoxynucleotidyl transferase-mediated dUTP nick end-labeling; UUO, unilateral uretal obstruction.

To further examine the renoprotective mechanisms of KMSC-derived MPs, the degree of renal cell proliferation and microvascular density were examined in the kidneys of control, KMSC, MPs, and MP-RNase injected mice. The total number of PCNA-positive renal cells was significantly greater in mice kidneys injected with KMSC and KMSC-derived MPs than kidneys injected with vehicle or MP-RNase (34.7 ± 6.1, 42.8 ± 4.4 *versus* 15.8 ± 2.9, 24.3 ± 3.0 per field, respectively, *P <0.05) (Figure 5A, B). The degree of renal microvascular rarefaction assessed by CD31 expression was significantly less in mice injected with KMSC and KMSC-derived MPs than in control or MP-RNase injected mice (9.8 ± 1.6, 15.3 ± 2.9 *versus* 48.8 ± 4.1, 44.7 ± 4.2 per field, respectively, *P <0.05) (Figure 5A, B).

The anti-apoptotic effects of MPs on UUO injury were evaluated by TUNEL staining. At day 7 after UUO injury, there were significantly fewer TUNEL positive apoptotic nuclei in UUO kidneys injected with either KMSC or KMSC-derived MPs than those injected with vehicle or RNase-treated MPs (0.4 ± 0.3, 1.4 ± 0.5 *versus* 55.4 ± 5.7, 64.6 ± 5.1 cells per HPF respectively, *P <0.05) (Figure 5A, B). Apoptotic PTC endothelial cells coexpressed CD31 and TUNEL. The total number of TUNEL positive PTC endothelial nuclei was also significantly reduced in UUO kidneys treated with KMSC or KMSC-derived MPs compared to mice injected with vehicle control or RNase-treated MPs (0.2 ± 0.2, 1.0 ± 0.6 *versus* 13.8 ± 1.2, 11.8 ± 0.6 cells per HPF, respectively, *P <0.05) (Figure 5A, B).

**Figure 6:**
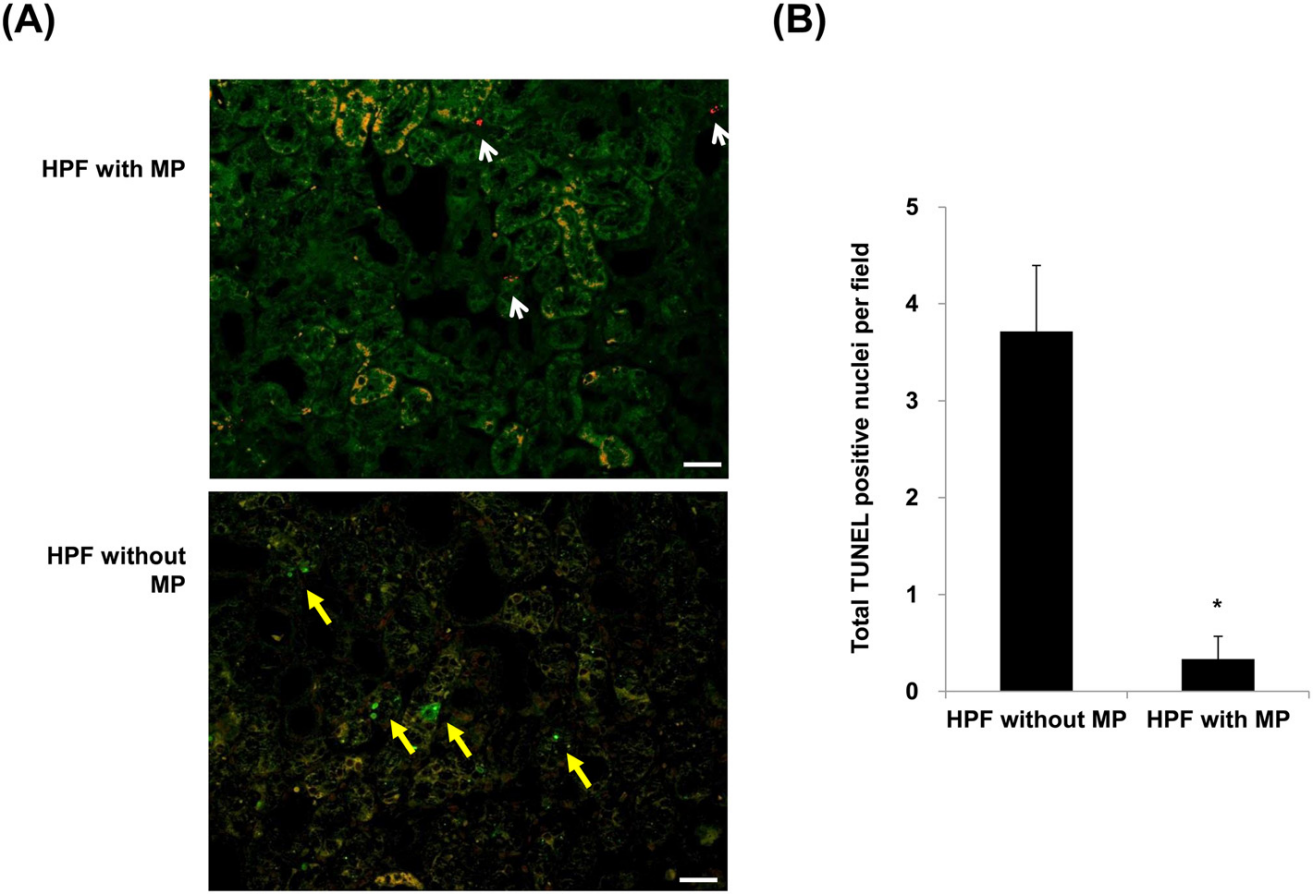
**Anti-apoptotic effects of** ***in vivo*** **engrafted-MPs in UUO kidneys.** Confocal laser microscopy confirms the engraftment of red CellTracker™ labeled MPs and TUNEL staining in UUO kidneys. High-power fields in which red CellTracker™ labeled MPs (white arrows) were present showed no apoptotic cells (TUNEL positive green nuclei, yellow arrows), compared to high-power fields without MPs **(A)**. Quantitative analysis demonstrates the total number of TUNEL-positive nuclei in high-power fields with or without MPs. Results are expressed as the mean ± SE of six different experiments. The Kruskal-Wallis test was performed; * *P* <0.05 versus vehicle control **(B)**. White bar represents 20 μm. MPs, microparticles; SE, standard error; TUNEL, terminal deoxynucleotidyl transferase-mediated dUTP nick end-labeling; UUO, unilateral uretal obstruction.

We next investigated whether KMSC-MPs demonstrated local anti-apoptotic effects in UUO kidneys. Red CellTracker™ labeled MPs were infused into UUO mice immediately after the procedure, and we used confocal microscopy to analyze kidney regions in UUO mice in which red CellTracker™ labeled MPs were present or not for apoptotic cells shown by TUNEL staining. Regions containing red CellTracker™ labeled MPs (white arrows) did not show apoptotic cells (green nuclei, yellow arrows), compared to regions without red CellTracker™ labeled MPs, thereby suggesting that these regions were protected (Figure 6A). By semi-quantification analysis, TUNEL-stained kidneys at high-power fields (×20) showed a significantly lower number of apoptotic cells in fields containing MPs, compared to fields without MPs (0.3 ± 0.2 *versus* 3.7 ± 0.7 cells per HPF, respectively, *P* <0.05) (Figure 6B).

### *In vivo* effect of microparticles on EndoMT in UUO kidneys

**Figure 7:**
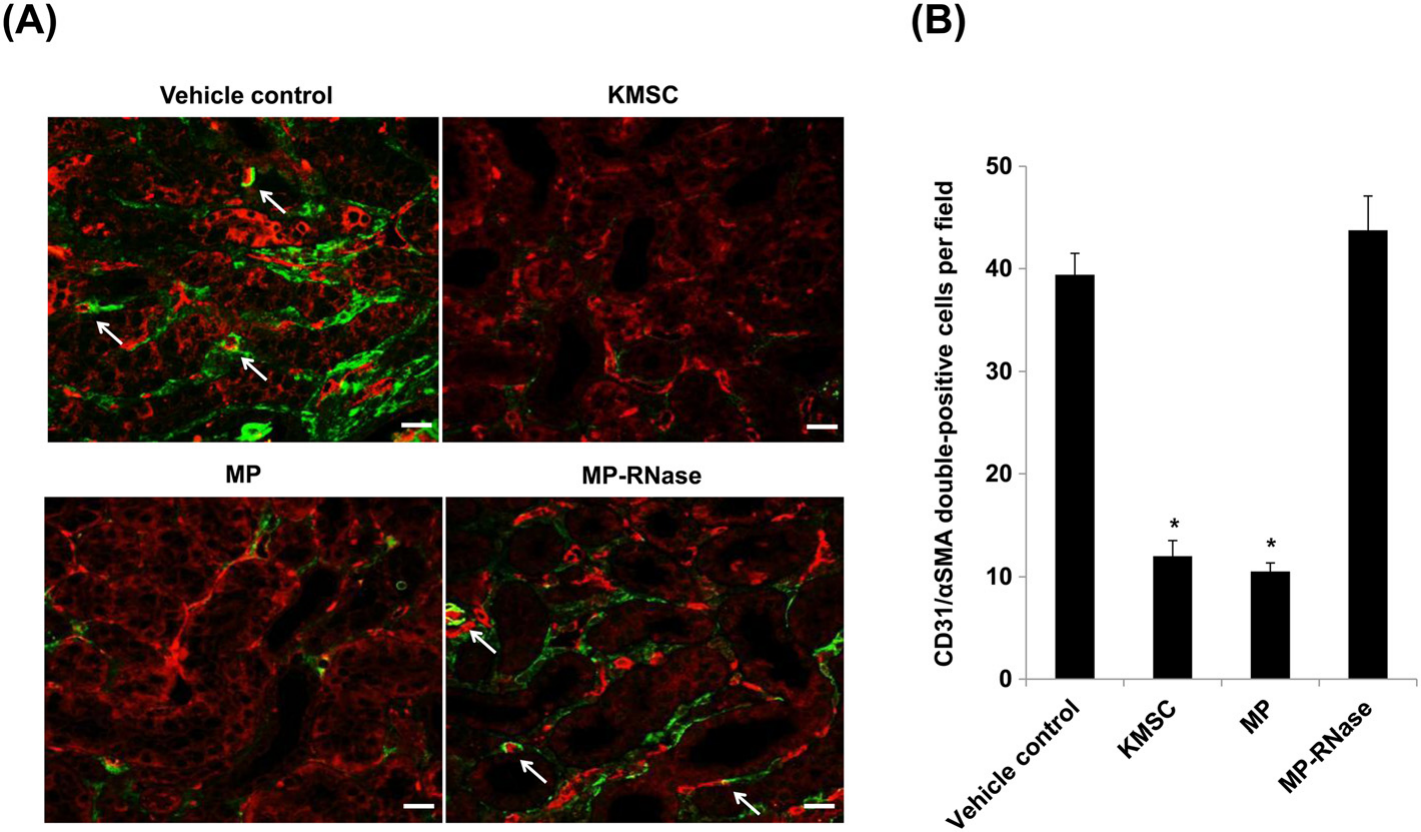
**Endothelial-to-mesenchymal transition in UUO kidney.** Confocal laser microscopy analysis shows representative images of CD31 and α-SMA double-staining in UUO kidneys treated with vehicle control, KMSC, KMSC-derived MP, and MP-RNase (**A**, CD31; red color, α-SMA; green color). Quantitative analysis of CD31 and α-SMA double-positive cells per field (white arrows) in UUO kidneys. Results are expressed as the mean ± SE of six different experiments. The Kruskal-Wallis test was performed; * *P* <0.05 versus vehicle control **(B)**. White bar represents 20 μm. KMSC, kidney-derived mesenchymal stem cells; MPs, microparticles; SE, standard error; UUO, unilateral uretal obstruction; α-SMA, α-smooth muscle actin.

Next, we performed double staining for α-SMA positive fibroblasts with CD31 to show whether KMSC and KMSC-derived MPs exert *in vivo* effects on EndoMT in UUO kidneys. Confocal microscope analysis revealed the existence of α-SMA positive fibroblasts with CD31 in UUO kidneys treated with vehicle control or MP-RNase. By semi-quantitative analysis, the number of α-SMA positive fibroblasts with CD31 was significantly less in mice kidneys injected with KMSC and KMSC-derived MPs than mice injected with vehicle or MP-RNase (12.0 ± 1.5, 10.5 ± 0.8 versus 39.4 ± 2.1, 43.8 ± 3.4 cells per HPF, respectively, *P <0.05) (Figure 7A, B). These findings suggested that administration of KMSC and KMSC-derived MPs in UUO kidneys offers beneficial effects on UUO injury via inhibition of EndoMT of PTC endothelial cells.

## Discussion

In the present study, we demonstrated that MPs administered following UUO localized within PTC and these MPs significantly decreased EndoMT, enhanced endothelial cell proliferation and reduced apoptosis that resulted in decreased rarefaction of PTC. Administration of MPs also inhibited infiltration of inflammatory macrophages (F4/80 positive) and decreased tubulointerstitial fibrosis. MPs preincubated with RNase did not show the same protective effects, suggesting that the renoprotective effects of the MPs were mediated in a manner at least partially dependent on mRNA transfer to target cells.

We recently demonstrated that MPs derived from KMSCs cultured in anoxic conditions express high levels of proangiogenic VEGF-A mRNA. When these MPs were administered following acute ischemia-reperfusion, the MPs localized into tubular cells as well as PTC endothelial cells, and resulted in significant amelioration of PTC rarefaction and improvement in renal function [13]. Similarly, the present study showed that KMSC-derived MPs stained with CellTracker™ engrafted in PTC in UUO kidneys and the administration of KMSC and KMSC-derived MPs improved renal microvascular rarefaction and endothelial cell proliferation. Moreover, apoptosis of not only tubular epithelial cells but also PTC endothelial cells was significantly reduced by injection of KMSCs or KMSC-MPs into UUO mice, and this resulted in preservation of the peritubular microvasculature. Interestingly, *in vivo* homing and engraftment of KMSC-derived MPs was only observed in obstructed kidneys. This suggests that homing and engraftment of MPs into injured tissue also operates in an injury signal-induced fashion. The exact mechanism of homing and engraftment of MPs into injured tissue has not been addressed in the present study. However, as MPs have comparable surface molecules as their cell of origin, it would not be wrong to speculate that they would involve adhesion molecules and ligands such as CD44, CD29, α4-, α5-, and α6-integrins [13]. Importantly, analysis of renal cortex and corticomedullary areas surrounding engrafted MPs showed significantly lower numbers of apoptotic cells, compared with areas that did not have MPs. This suggests that MPs deliver anti-apoptotic signals into adjacent renal cells and possibly promote their cellular survival. Similar results have been demonstrated previously by Togel *et al*. in a rat model of ischemic injury, where administration of bone marrow MSCs homed to ischemic kidneys, and renal cells in the vicinity of MSC showed less apoptosis [21]. Nevertheless, the extent to which MPs are incorporated into target cells is only 5 to approximately 20/mm^2^ of individual kidney sections and the exact mechanism by which this small number of MPs can induce reprogramming of their target cells is unknown. Furthermore, studies should be performed to outline how MPs migrate from the blood through the basal membrane into target cells in injured kidneys.

To date, little is known of the exact mechanism for inducing PTC endothelial cell loss. One possible mechanism that has been intensively investigated is EndoMT, which would increase fibroblast population while decreasing endothelial cells. Endothelial lineage tracing, using Tie2-Cre;R26R-stop-EYFP transgenic mice, confirmed the presence of up to 36% of EndoMT-derived fibroblasts in UUO nephropathy [8]. In the present study, we demonstrated that MPs significantly inhibited EndoMT of TGF-β1-treated HUVEC. This effect was mediated by incorporation of KMSC-derived MPs and the resulting mRNA transfer into target HUVEC as RNase pretreated MPs failed to show any beneficial effect. Furthermore, administration of KMSC-derived MPs immediately following UUO significantly ameliorated EndoMT of PTC endothelial cells and decreased the PTC rarefaction index. Confocal analysis showed that α-SMA positive fibroblasts co-expressing CD31 were significantly decreased in UUO mice injected with KMSC-derived MPs. To the best of our knowledge, the present study is the first to demonstrate that KMSC-derived MPs can inhibit EndoMT *in vitro* and *in vivo*. Enhanced PTC endothelial cell proliferation or reduced endothelial cell apoptosis is another mechanism by which KMSC-derived MPs may improve preservation of PTC in UUO mice. Our previous study demonstrated that incubation of HUVEC with KMSC-derived MPs promotes a significant dose-dependent improvement in HUVEC proliferation in serum deprived culture conditions [13]. In accordance with our previous data, KMSC-derived MPs significantly improved *in vitro* TGF-β1-treated HUVEC, partly mimicking the microenvironment of PTC in UUO mice kidneys. Thus, our present data are in strong support of the notion that PTC rarefaction can be significantly ameliorated upon treatment with KMSC-derived MPs. Morphometric analysis revealed that KMSC-derived MPs reduced interstitial infiltration of F4/80 positive macrophages or α-SMA positive (myo)fibroblasts and improved renal fibrosis. Renal fibroblast proliferation and extracellular matrix production are driven in part by renal ischemia [22]. The administration of KMSC-derived MPs into UUO mice, via transfer of proangiogenic signals such as VEGF-A, promoted endothelial cell proliferation and decreased EndoMT, resulting in significant increases in the number of PTC. This preservation of PTC density in UUO kidneys may have contributed to decreased intrarenal hypoxia and ameliorated inflammatory cell infiltration and subsequent tubulointerstitial fibrosis [8].

One limitation of the present study is that, although PTC as a whole showed increased density and less EndoMT in UUO mice treated with KMSC-derived MPs, we have no direct data to demonstrate the fate of PTC endothelial cells that were engrafted with KMSC-derived MPs. Such analysis would require cell fate tracing experiments. Notwithstanding, *in vitro* HUVEC experiments showed definite internalization of PKH26 labeled MPs into HUVEC and their proangiogenic effects. In addition, kidney cells in the vicinity of engrafted MPs showed significantly lower numbers of apoptotic cells suggesting that MPs deliver anti-apoptotic signals into adjacent renal cells to promote their cellular survival. This ‘paracrine’ effect, therefore, could not be assessed by morphometric analysis of surface labeled MPs. Another limitation is that expression of various proangiogenic or anti-angiogenic molecules in the UUO kidneys were not investigated after administration of KMSC-derived MPs. VEGF-A, a predominant regulator of angiogenesis, has been implicated as an important mediator in preserving PTC in chronic kidney injury. In a rat model of UUO, Ohashi *et al*. observed early PTC endothelial cell proliferation that was accompanied by intense expression of VEGF within the tubular epithelium [6]. In line with this, deprivation of proangiogenic growth factors (for example, VEGF) induced endothelial cell apoptosis. However, multiple studies report discrepancies, especially the optimal time period of VEGF-A action and the expression thereof in chronic kidney disease. Our previous studies demonstrated that administration of KMSC-derived MPs and KMSC into an acute ischemic injury mouse model effectively delivered VEGF-A mRNA and increased intrarenal VEGF-A expression up to five-fold, respectively [13]. As the focus of the present study was to investigate the role of KMSC-derived MPs in EndoMT and their role in protecting PTC density, investigation of expression of various angiogenic molecular targets in UUO mice treated with KMSC-derived MPs would need a separate set of experiments. Another limitation is that Masson’s trichrome staining was used for the assessment of kidney fibrosis in UUO kidneys. As trichrome stains basement membranes and brush borders, the thickened or edematous basement membranes of tubules might also be stained and thereby assessed as kidney fibrosis [23].

Finally, the present study showed amelioration of EndoMT in cultured HUVEC which exhibited the incorporation of red PKH26-labeled MPs. However, we did not analyze *in vivo* EndoMT of endothelial cells engrafted with MPs in UUO mice in comparison to those that were not. Therefore, the present study might have underestimated the beneficial effects of KMSC-derived MPs on EndoMT in UUO kidneys. Also, since we assessed *in vivo* EndoMT in cells only co-expressing α-SMA and CD31 markers, we might have missed cells that had already lost endothelial markers [8].

## Conclusions

In summary, KMSC-derived MPs showed renoprotective effects by ameliorating renal fibrosis, inflammation, and EndoMT and preserving PTC endothelial cells in UUO kidneys. Our data suggest that KMSC-derived MPs could be a potential therapeutic approach against chronic renal fibrosis.

## Abbreviations

EndoMT: endothelial-to-mesenchymal transition
HPF: high-power field
HUVEC: human umbilical vein endothelial cell
KMSC: kidney-derived mesenchymal stem cell
MPs: microparticles
PCNA: proliferating cell nuclear antigen
PTC: peritubular capillaries
TGF-β1: transforming growth factor-β1
TUNEL: terminal deoxynucleotidyl transferase-mediated dUTP nick end-labeling
UUO: unilateral ureteral obstruction
VEGF: vascular endothelial growth factor
α-SMA: α-smooth muscle actin
